# Serum Selenium Status as a Diagnostic Marker for the Prognosis of Liver Transplantation

**DOI:** 10.3390/nu13020619

**Published:** 2021-02-14

**Authors:** Safak Gül-Klein, Deana Haxhiraj, Julian Seelig, Anika Kästner, Julian Hackler, Qian Sun, Raban Arved Heller, Nils Lachmann, Johann Pratschke, Moritz Schmelzle, Lutz Schomburg

**Affiliations:** 1 Department of Surgery, Charité Campus Mitte and Campus Virchow Klinikum, Charité-Universitätsmedizin Berlin, Corporate Member of Freie Universität Berlin, Humboldt-Universität zu Berlin, and Berlin Institute of Health, 13353 Berlin, Germany; safak.guel@charite.de (S.G.-K.); anika.kaestner@charite.de (A.K.); johann.pratschke@charite.de (J.P.); moritz.schmelzle@charite.de (M.S.); 2 Institute for Experimental Endocrinology, Charité-Universitätsmedizin Berlin, Corporate Member of Freie Universität Berlin, Humboldt-Universität zu Berlin, and Berlin Institute of Health, 10115 Berlin, Germany; deah97@zedat.fu-berlin.de (D.H.); julian.seelig@charite.de (J.S.); julian.hackler@charite.de (J.H.); qian.sun@charite.de (Q.S.); raban.heller@tutanota.com (R.A.H.); 3 Department of General Practice and Health Services Research, University Hospital Heidelberg, 69120 Heidelberg, Germany; 4 Institute of Transfusion Medicine, Charité-Universitätsmedizin Berlin, Corporate Member of Freie Universität Berlin, Humboldt-Universität zu Berlin, and Berlin Institute of Health, 13353 Berlin, Germany; nils.lachmann@charite.de

**Keywords:** trace element, liver transplantation, selenoprotein P, glutathione peroxidase, hepatitis C virus

## Abstract

The trace element selenium (Se) is taken up from the diet and is metabolized mainly by hepatocytes. Selenoprotein P (SELENOP) constitutes the liver-derived Se transporter. Biosynthesis of extracellular glutathione peroxidase (GPx3) in kidney depends on SELENOP-mediated Se supply. We hypothesized that peri-operative Se status may serve as a useful prognostic marker for the outcome in patients undergoing liver transplantation due to hepatocellular carcinoma. Serum samples from liver cancer patients were routinely collected before and after transplantation. Concentrations of serum SELENOP and total Se as well as GPx3 activity were determined by standardized tests and related to survival, etiology of cirrhosis/carcinoma, preoperative neutrophiles, lymphocytes, thyrotropin (TSH) and Child–Pugh and Model for End-Stage Liver Disease (MELD) scores. A total of 221 serum samples from 79 transplanted patients were available for analysis. The Se and SELENOP concentrations were on average below the reference ranges of healthy subjects. Patients with ethanol toxicity-dependent etiology showed particularly low SELENOP and Se concentrations and GPx3 activity. Longitudinal analysis indicated declining Se concentrations in non-survivors. We conclude that severe liver disease necessitating organ replacement is characterized by a pronounced Se deficit before, during and after transplantation. A recovering Se status after surgery is associated with positive prognosis, and an adjuvant Se supplementation may, thus, support convalescence.

## 1. Introduction

The liver is of central relevance for trace element metabolism, in particular for controlling copper, iron, selenium (Se) and zinc homoeostasis [1,2,3]. Under inflammatory conditions, several converging pathways contribute to declining serum Se status, in part via reduced biosynthesis of the Se transport protein selenoprotein P (SELENOP) in hepatocytes as part of the negative acute phase response (APR) [4,5,6,7]. Studies in lipopolysaccharide (LPS)-injected mice as a rodent model of APR have indicated that the inflammatory stimulus reduces the transcription of central genes controlling Se metabolism, selenocysteine (Sec) formation and Sec insertion into growing selenoproteins [8,9,10]. Among the rate-limiting hepatic factors are the intracellular Se-binding protein 1 (Selenbp1), the phosphorseryl-tRNA-kinase (Pstk) and the Sec-specific elongation factor (eEFsec) [5,11]. Collectively, an impaired hepatic Se metabolism caused by an APR is associated with decreased SELENOP biosynthesis and secretion, causing a declining Se status in the circulation and in target tissues, as observed, e.g., in sepsis [12], severe trauma [13], inflammatory disease [14] or in COVID-19 [15].

The central role of the liver for an undisturbed Se metabolism and Se status within normal reference ranges is supported by analyses of patients with liver disease [1,16,17,18]. Cirrhotic or fibrotic liver tissue causes reduced serum Se concentrations, and a recent report indicated a gradual decrease of serum SELENOP concentrations in non-alcoholic fatty liver disease (NAFLD) and steatohepatitis (NASH) [19]. The essential nature of Se for liver health was first proven in a rodent model of tissue necrosis in 1957 by Schwarz and Foltz, where supplemental Se proved essential for preventing organ destruction [20,21]. The direct interplay of Se and vitamin E in relation to iron-mediated oxidative stress and damage is of central importance for ferroptosis, a general mechanism of cell death [22,23]. The liver is constantly exposed to high oxidative stress, as it decisively contributes to the safe removal of endogenous and exogenous toxicants. Consequently, it was postulated that insufficient Se supply and low expression of relevant selenoproteins implicated in antioxidative protection might contribute to a higher risk of malignant transformation of hepatocytes, and hence, higher incidences of liver tumors [24,25]. This notion was supported by a focused epidemiological case-control study nested within the European Prospective Investigation into Cancer and Nutrition (EPIC) cohort, where Se and SELENOP deficiency, respectively, were identified as the most relevant risk factors for hepatocellular carcinoma (HCC) [26]. A Se or SELENOP concentration within the lowest tertile of the European population was associated with a 5- to 10-times increased risk for HCC [27].

A similarly close interrelation is described for Se deficits in viral infection and autoimmune disease. Patients suffering from hepatitis C virus (HCV) or hepatitis B virus (HBV) infection are characterized by relatively low Se status [28,29,30]. Another direct connection between Se and liver disease is given by the O-phosphoseryl-tRNA:selenocysteinyl-tRNA synthase (SEPSECS) [31], which has initially been described as soluble liver antigen SLA/LP in a distinct form of hepatic autoimmune condition [32,33].

From all of the above, we hypothesized that patients with severe liver disease undergoing liver transplantation are characterized by severe dysregulation of Se metabolism, and that one or all of the Se status biomarkers accessible from serum samples provide relevant and useful information for tissue functioning, transplantation success and prognosis.

## 2. Materials and Methods

### 2.1. Study Design

**Figure 1:**
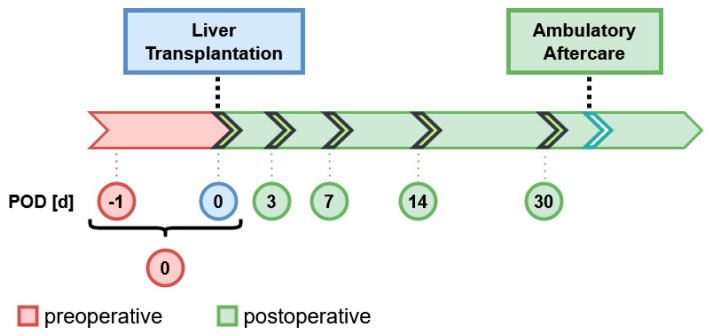
Sampling scheme for patients undergoing liver transplantation (LT). The first serum sample was taken at day of LT, including a time frame of 24 h before, designated as postoperative day 0 (POD 0). Consecutive samples post-LT were collected on the 3rd, 7th, 14th and 30th day post-LT (POD 3, 7, 14, 30).

A longitudinal study of patients with hepatocellular carcinoma (HCC) that were selected for liver transplantation (LT) was conducted at the Department of Surgery at Charité—Universitätsmedizin Berlin. All patients analyzed received cadaveric organ transplants and had provided written informed consent for participation in the study. The samples were collected between March 2009 and December 2015 and were deposited in a local biobank at −80 °C according to a predefined scheme (Figure 1). Routine diagnostic clinical parameters were determined as described [34], and follow-up over a time span of five years was performed. Samples were analyzed retrospectively in 2020 at the Institute for Experimental Endocrinolgy, Charité Berlin. According to a predefined scheme, the available samples were assigned to specific time points, yielding a final set of *n* = 221 serum samples from *n* = 79 transplanted patients (Supplementary Figure S1). The study was conducted in accordance with the declaration of Helsinki. Approval was granted by the Board of Ethics of Charité Medical School Berlin (EA2/092/19). All measurements were conducted by scientists blinded to any clinical information.

### 2.2. Selenium Status Analysis

Concentration of total serum Se was determined by total reflection X-ray fluorescence (TXRF) analysis using a benchtop TXRF analyzer (T-Star, Bruker Nano GmbH, Berlin, Germany) as described [35]. Seronorm serum standard (Sero AS, Billingstad, Norway) served as control, and inter- and intra-assay coefficients of variation (CV) of the measurements were <5%. Concentrations of selenoprotein P (SELENOP) were determined by a commercial immunometric sandwich assay (selenOtest ELISA, selenOmed GmbH, Berlin, Germany). Internal assay kit controls were measured in duplicate in concentration steps of 18.2 to 72.9 and up to 291.7 µg/mL, yielding inter- and intra-assay CV ≤ 12.8%. Serum glutathione peroxidase (GPx3) activity was measured using tert-butyl-hydroperoxide as substrate as described recently [36]. A pool of 25 human serum samples (commercial samples, provided by invent Diagnostica GmbH, Hennigsdorf, Germany) served as a control for GPx3 measurements, yielding inter- and intra-assay CV ≤ 11.0%.

### 2.3. Statistical Analysis

All statistical calculations were performed with R version 4.0.3, applying the packages “tidyr,” “dplyr” and “pROC” [37]. Figures were created by using the package ”ggplot2” [38]. Correlations were tested by Spearman correlation analysis. Comparisons of the characteristics between two groups were conducted by Mann–Whitney U test, more than two groups were compared with a Kruskal–Wallis test.

As this is an exploratory post hoc analysis, all *p*-values are to be interpreted descriptively, and no adjustment for multiple testing was adopted. Variable selection was performed via stepwise AIC selection [39,40]. Differences between ROC curves were assessed by the DeLong’s test for two correlated ROC curves [41]. All statistical tests are using an α-level of 0.05; * *p* < 0.05, ** *p* < 0.01, *** *p* < 0.001 and **** *p* < 0.0001.

## 3. Results

### 3.1. Selenium Status

Se status of patient samples was evaluated by different biomarkers, including total serum Se and serum SELENOP concentrations, for which reference data are available from a large cross-sectional study (EPIC; the European Prospective Investigation into Cancer and Nutrition) [42], as well as GPx3 activity, for which reference data are not available. A high fraction of the serum samples from the LT patients displayed a considerable Se deficit at the time of surgery with median Se and SELENOP concentrations of 55.4 µg/L and 2.06 mg/L, respectively (Table 1).

**Figure 2:**
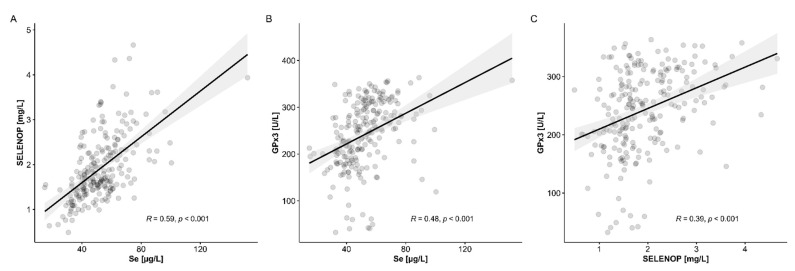
Interrelation of the biomarkers of Se status in the samples from patients undergoing LT. (**A**) Total serum Se and SELENOP concentrations showed a tight and linear correlation over the full range of data (*R* = 0.59). (**B**) Similarly, extracellular GPx3 activity correlated positively and linearly to serum Se concentrations (*R* = 0.48). (**C**) The two circulating protein biomarkers of Se status, i.e., SELENOP and GPx3, showed a moderate positive correlation in the samples analyzed (*R* = 0.39); *n* = 221, *R*; Spearman correlation coefficient (two-sided, two-tailed).

Considering the full set of samples available for analysis, i.e., from the time point of surgery (POD 0) up to 30 days post LT (POD 30), correlation analyses of total serum Se and SELENOP as well as GPx3 activity were conducted (Figure 2A–C). The results obtained support the notion of a general deficiency in the trace element Se as stringent correlations were observed between all of the three biomarkers, highlighting at the same time the high quality and integrity of the clinical samples available for analysis. As known from similar clinical analyses, the most stringent correlation is observed between total serum Se with SELENOP (Figure 2A), followed by GPX3 with Se (Figure 2B) and GPX3 with SELENOP (Figure 2C).

### 3.2. Comparison of Se Status in Relation to Reference Ranges and HCC Etiology

**Figure 3:**
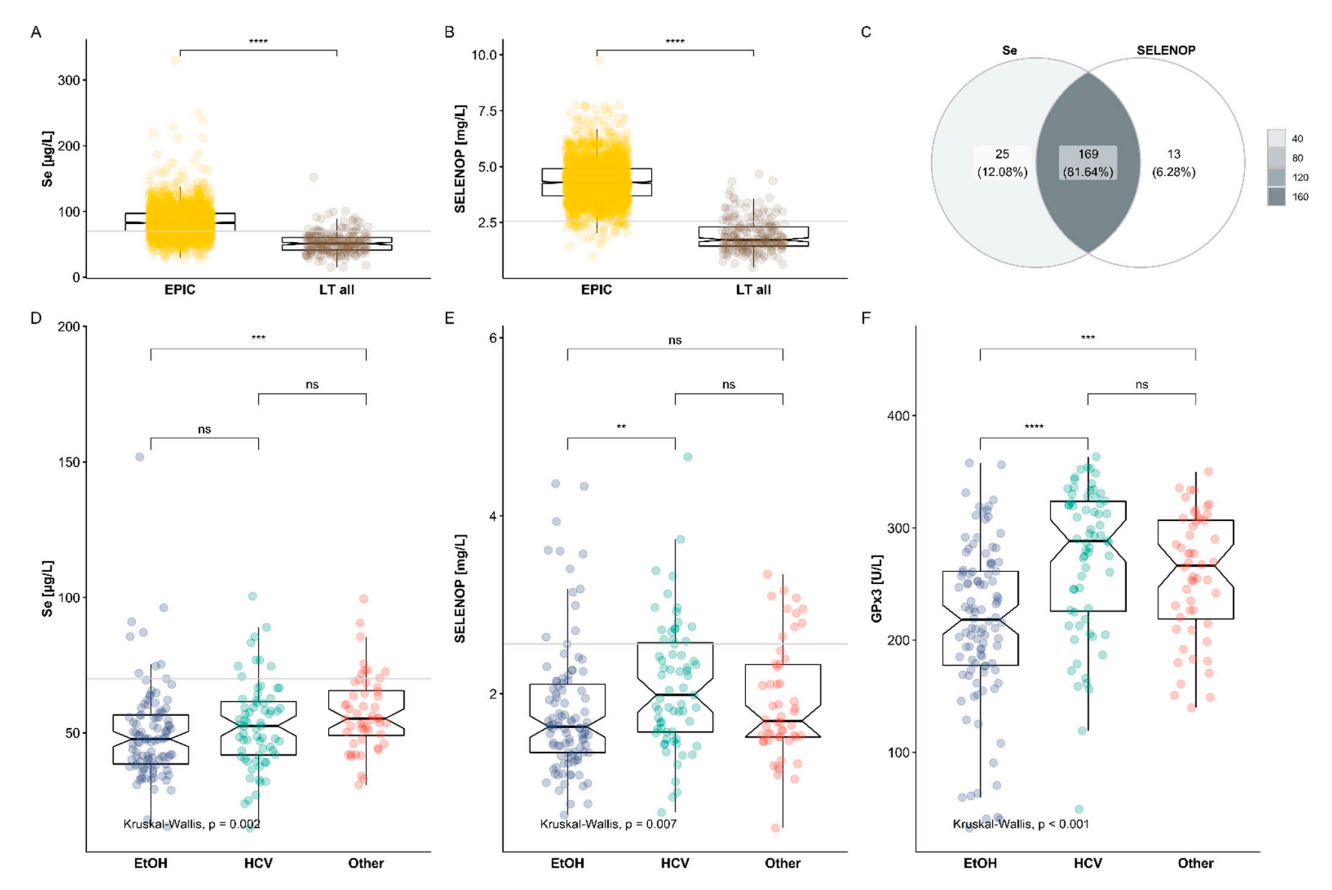
Depressed Se status in patients undergoing liver transplantation (LT). The set of serum samples from patients undergoing LT displayed a general Se deficit, with very low concentrations of (**A**) total Se and (**B**) SELENOP in comparison to a reference cohort of healthy subjects (EPIC). The thresholds for total Se and SELENOP deficiency, i.e., below 70 µg/L and 2.56 mg/L, respectively, are indicated by grey horizontal lines (**A**,**B**,**D**,**E**). In relation to these criteria, (**C**) the majority of patient samples (207/221, 93.7%) displayed a deficit in serum Se or SELENOP, and 81.6% of these presented with a combined deficiency. (**D**–**F**) The samples were subdivided according to the etiology of hepatocellular carcinoma (HCC) in liver cirrhosis. The largest group represented the patients with cirrhosis due to an ethanol toxicity (EtOH, *n* = 102, 37 patients), followed by patients with hepatitis C virus cirrhosis (HCV, *n* = 67, 25 patients) and a group with other etiologies (*n* = 52, 17 patients). The patients with EtOH-dependent etiology showed relatively low (**D**) total serum Se and (**E**) SELENOP concentrations as well as (**F**) particularly low GPx3 activities. Comparisons by Kruskal–Wallis test; ** *p* < 0.01, *** *p* < 0.001 and **** *p* < 0.0001.

The Se status determined by total serum Se and circulating SELENOP concentrations was compared to the reference ranges of a large cohort of healthy European subjects analyzed by the same technology in the same analytical lab. These reference data were derived from subjects participating in the EPIC cohort, where GPx3 activity had not been determined [42]. Mean serum Se concentrations of the patients undergoing LT differed significantly from reference values (Figure 3A). The average Se concentration observed (52.5 µg/L) was in the range of the 6th percentile of healthy subjects in EPIC (53.2 µg/L). SELENOP concentrations were also significantly lower than the reference group, and mean SELENOP concentrations in LT samples (1.9 ± 0.7 mg/L) were even below the 1st percentile of the EPIC cohort (2.3 mg/L) (Figure 3B). The vast majority of samples displayed a profound deficit in either Se or SELENOP (93.7%), and most of these samples displayed a combined deficit (81.6%) with respect to the thresholds for Se deficiency at 70 µg/L and SELENOP at 2.56 mg/L [43] (Figure 3C).

Next, the liver patients were divided into three groups in relation to etiology of liver cirrhosis, i.e., patients with ethanol-induced (EtOH) cirrhosis (*n* = 37), HCV-induced cirrhosis (*n* = 25) or other etiology (*n* = 17). A direct comparison revealed particularly low Se status in patients with EtOH-dependent etiology, both in relation to serum Se (Figure 3D) and SELENOP concentrations (Figure 3E) as well as in relation to GPx3 activities (Figure 3F).

### 3.3. Se Status Biomarkers in Relation to Survival

**Figure 4:**
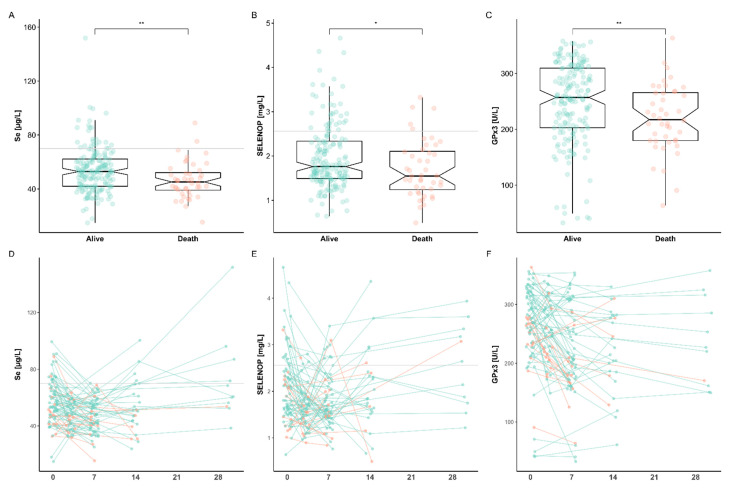
Comparison of Se status biomarkers in relation to survival. Patients undergoing liver transplantation (LT) were separated into survivors (Alive) and non-survivors (Death). Non-survivors displayed significantly reduced (**A**) serum Se, (**B**) SELENOP and (**C**) GPx3 levels as compared to survivors. A time resolved analysis of the individual patients (**D**–**F**) revealed consistently reduced concentrations of all three biomarkers in the majority of samples from postoperative day 0 (POD 0) to POD 7, and until POD 30. A general tendency of recovering Se status with time after surgery is noted in the majority of surviving patients, and in a minority of the non-survivors. Comparisons by Mann–Whitney U test; * *p* < 0.05, and ** *p* < 0.01.

Liver transplantation (LT) bears a certain risk of death, and 16 (20.3%) out of the 79 patients did not survive the intervention during a follow-up time of five years. The survival period of the deceased patients was at least 20 months, excluding acute postsurgical complications as a reason for mortality. Instead, tumor recurrence and infections, respectively, were the major causes of death. The depressed Se status biomarkers during surgery and the post-transplantation period were, therefore, compared and tested for their predictive value in relation to survival. The direct comparison of total serum Se and SELENOP concentrations along with GPx3 activities indicated a significant difference between patients who died within the follow up period (Death; group of non-survivors) and the patients surviving LT (Figure 4A–C). All three markers were significant higher in the group of survivors as compared to the group of non-survivors (Se [µg/L]; 54.1 ± 16.1 vs. 46.5 ± 13.4; SELENOP [mg/L]; 2.0 ± 0.7 vs. 1.7 ± 0.6; GPx3 [U/L]; 247.9 ± 72.9 vs. 219.6 ± 59.6).

Time-resolved analyses indicated a considerable heterogeneity of the initial levels and different dynamics of the Se status between the individuals with regard to outcome (Figure 4D–F). Total serum Se declined initially to a similar degree in both groups of patients, but seemed to recover in survivors soon after LT (Figure 4D). In comparison, circulating SELENOP and GPx3 activities were similar in both groups, with non-survivors displaying a tendency of stronger deficits (Figure 4E,F).

### 3.4. Comparison of Se Status Biomarkers with Established Indices of Chronic Liver Disease

**Figure 5:**
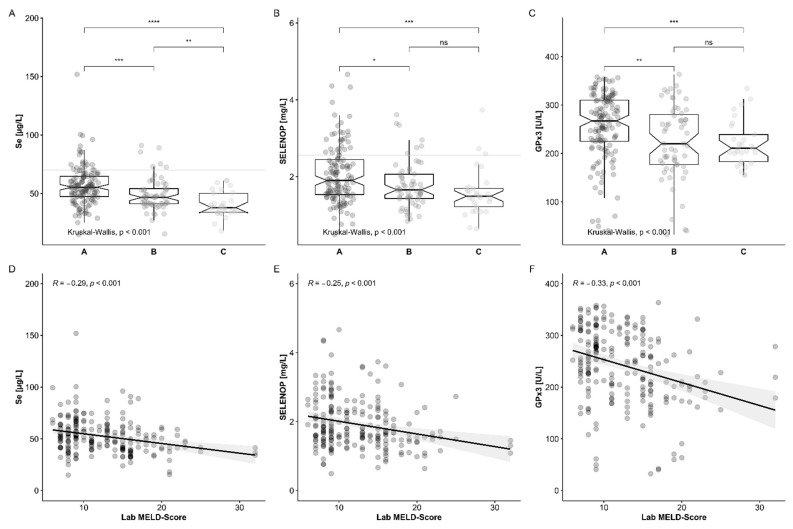
Serum biomarkers of Se status from patients undergoing liver transplantation (LT) at the time of surgery (POD 0) in comparison to established diagnostic scoring systems. All three biomarkers of Se status, i.e., (**A**,**D**) total serum Se concentrations, (**B**,**E**) SELENOP levels and (**C**,**F**) GPx3 activities, showed a significant relation to the Child–Pugh Score (CPS) and the MELD score. The most severe diagnosis (CPS class C) displayed the lowest (**A**) Se concentrations, (**B**) SELENOP levels and (**C**) GPX3 activities. Similarly, the correlation coefficients of the Se biomarkers with the MELD score were consistently negative, and ranged from (**D**) R = −0.29 for total Se, to (**E**) R = −0.25 for SELENOP levels and to (**F**) R = −0.33 for GPX3 activities. Comparisons between groups by Kruskal–Wallis test (**A**–**C**), and correlation analyses by Spearman analysis; * *p* < 0.05, ** *p* < 0.01, ** *p* < 0.001 and **** *p* < 0.0001.

In order to assess the potential value of the Se status biomarkers as additional diagnostic parameters of liver disease, the initial data at the time of surgery (POD 0) were compared to the established indices of chronic liver disease (Figure 5). Total serum Se concentrations correlated to liver disease severity as assessed by the Child–Pugh Score (CPS), and patients with the most severe diagnosis (CPS class C) displayed the lowest Se levels (Figure 5A). The same interrelation is observed for the other two biomarkers of Se status, i.e., for SELENOP concentrations (Figure 5B) and GPx3 activities (Figure 5C), respectively.

In a second analysis, the Se status was compared to the lab MELD score. All three biomarkers of Se status determined at time of surgery (POD 0) were related to the MELD score (Figure 5D–F). All three biomarkers correlated inversely (Spearman correlation coefficients R; two-sided, two-tailed), and the correlation coefficients ranged from R = −0.29 for total Se (Figure 5D), to R = −0.25 for serum SELENOP (Figure 5E) and to R = −0.33 for GPx3 activities (Figure 5F).

### 3.5. Diagnostic Value of Se Status for Predicting Survival after Liver Transplantation

**Figure 6:**
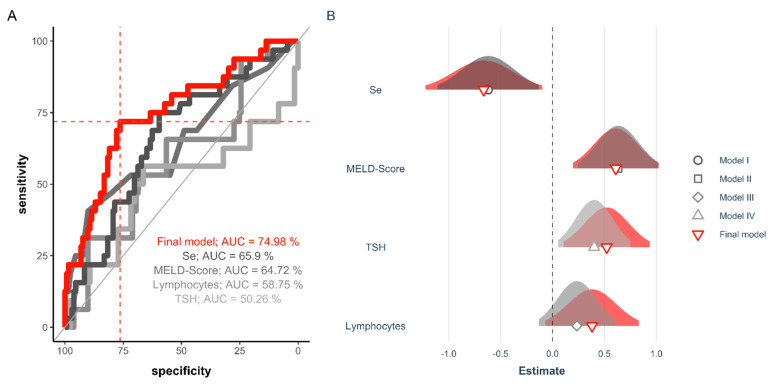
ROC analysis of serum biomarkers to predict non-survival after liver transplantation. The variable selection process included pre-OP TSH, pre-OP neutrophiles, pre-OP lymphocytes, the pre-OP neutrophiles/lymphocytes ratio (NLR), the MELD score, as well as longitudinal data concerning the GPx3 activity, CRP concentration, serum Se and SELENOP data. (**A**) Univariate assessments of total serum Se, the MELD score, pre-OP lymphocytes and pre-OP TSH predict survival of patients undergoing liver transplantation with a similar, albeit only poor to moderate, AUC. The multiple regression model based on the MELD Score, TSH and the lymphocytes at POD 0 together with the Se concentration outperformed any other combination of variables via stepwise AIC selection (final model; AUC = 75%; red line). The corresponding cutpoint according to the Youden Index is indicated in red. (**B**) The estimates of the final model are given alongside with the confidence intervals.

The patients undergoing LT were characterized by a notable Se deficiency in comparison to healthy adult subjects, with surviving patients displaying a better Se status than non-survivors. In order to assess the predictive value of Se status for survival, all three Se status biomarkers were tested separately and in different combinations with the other clinical parameters by receiver operating characteristic (ROC curve) analysis. The analyses indicated that total serum Se was of similar quality as the MELD score for predicting survival, yielding areas under the curve (AUC) of 65.9% and 64.7%, respectively (Figure 6A).

The final model based on Se, MELD score, the lymphocytes and TSH at POD 0 out-performed any other combination of variables (Se, SELENOP, GPX3, CRP, lymphocytes at POD 0, neutrophils at POD 0, the NLR at POD 0, the MELD score and TSH at POD 0) via stepwise AIC selection (Figure 6A). The final model, based on these four parameters, yielded an AUC of 75%. The corresponding cutpoint, according to the Youden Index [44] is indicated.

The specifics of the different models calculated indicate the relative quality of the individual parameters, and the estimate of the final model along with the corresponding confidence interval highlights its potential clinical value (Table 2).

## 4. Discussion

In this manuscript, we report the dynamic changes observed in Se status biomarkers in patients undergoing LT. The rationale for our analysis was based on the notion that hepatocytes are the key cell type for converting dietary Se into organic forms in the mammalian organism and for systemic transport of the trace element via SELENOP as the major selenoprotein in the circulation [45]. Transgenic mouse experiments have indicated that mice devoid of hepatic Selenop biosynthesis develop severe symptoms of Se deficiency, including growth defects, altered bone quality, impaired motor control and even epileptic seizures [6,46]. The brain phenotype was particularly unexpected, as the central nervous system constitutes a most preferentially supplied target organ in the hierarchy of Se distribution within the organism [47,48,49]. Notably, transgenic cell-specific expression of human SELENOP in hepatocytes only was capable of supplying the trace element into brain, thereby protecting the neurons from damage and death [50]. Based on these insights from model systems, the hypothesis of a strongly disturbed Se status in patients undergoing LT was tested and verified.

The results obtained support the findings from previous analyses in relation to a suppressed Se status in severe liver disease [16,51]. Chronic hepatitis, liver cirrhosis and hepatocellular carcinoma (HCC) are characterized by a depressed Se status [52]. Two underlying reasons may account for these strong and unequivocal findings; firstly, the loss of differentiated hepatocytes, and thereby an impaired uptake and conversion of the dietary trace element into circulating SELENOP [6,50], and secondly, a profound and intense local and systemic inflammation, known to negatively affect hepatic selenoprotein biosynthesis and depressed Se status in blood and likely in target tissues [50,53]. On top of these direct interrelations of impaired liver function and depressed Se status, a predisposition for severe liver disease, including HCC, may also contribute to the observed Se deficit in HCC, as shown in longitudinal and nested case-control studies [27,54].

All three biomarkers of Se status analyzed showed stringent correlations, supporting the assumption that a profound Se deficit is present in most if not all of the patients. Subjects with sufficiently high Se status are characterized by saturated circulating selenoprotein levels, with GPx3 activities reaching their plateau at around 80–90 µg Se per liter serum, and SELENOP showing a slightly higher requirement and reaching a plateau at 120–130 µg/L [55,56,57]. Under such Se replete conditions, a stringent correlation between total serum Se and GPx3 or SELENOP concentrations is not observed, indicating sufficient supply for maximized selenoprotein expression. The Se concentrations in our patients undergoing LT are far below these saturating concentrations, clearly supporting a classification as severely Se deficient, as also mirrored in the majority of samples residing below the consented threshold for Se deficiency (total serum Se < 70 µg/L and/or serum SELENOP < 2.56 mg/L). Under these conditions, expression of intracellular Se-dependent iodothyronine deiodinases may also be impaired, causing local or systemic hypothyroidism, which has been reported to constitute an independent risk factor for poor outcome after LT [58].

Further indications for the potential relevance of the perioperative Se status in liver transplantation became apparent when analyzing the predictive value of total serum Se in comparison to preoperative lymphocyte or neutrophile concentrations and their ratio (NLR). The NLR has been described as a valuable parameter reflecting the genesis and prognosis of HCC, suggesting that increased NLR is associated with poor outcome [59,60]. In our stepwise regression analysis, total serum Se had a better predictive value (AUC 65.9%) for poor outcome than lymphocytes (AUC 58.8%) or the NLR (AUC 51.1%). Unfortunately, thyroid hormone status was not systematically monitored in our group of patients and only preoperative TSH (pre-OP TSH) was available for analysis. Comparing these diagnostic parameters, the compound biomarker identified in the ROC curve analysis (Se, MELD score, pre-OP lymphocytes, pre-OP TSH) proved to be a promising tool for predicting non-survival after liver transplantation.

The central role of the liver for Se status is supported by the kinetic analyses of the Se status biomarkers. From the first postoperative day and during aftercare, our patients started with a low-germ diet without any specific Se-containing supplements. The Se status was low, remained low and even declined further during the first days after surgery. From the data obtained, recovery of SELENOP biosynthesis and secretion into the circulation seems to resume approximately one week post-LT. This time span may be needed for an initial recovery of liver function and hepatocyte metabolism, as blood supply improves and inflammatory tonus declines during the convalescence stage in surviving patients.

The kidneys are the major source of circulating GPx3, synthesized by renal tubular cells [61]. In transgenic mice, it was shown that renal GPx3 biosynthesis depends on hepatocyte-derived Selenop as source of the limiting trace element, mediated by a specific expression of the Selenop-receptor megalin (lipoprotein-related protein 2, Lrp2) [62,63]. This strict dependence of renal GPX3 on hepatic SELENOP may have contributed to the distinct but overlapping patterns of both circulating selenoproteins in the kinetic analysis of changes in Se status in the weeks after LT. How far a compromised kidney function with impaired GPx3 biosynthesis contributed to survival and prognosis remains to be studied in future analyses.

A universal, constant and massive Se deficit in patients with HCC undergoing LT remains as the major insight from this study. The deficit extents from the time before surgery, to the intervention and to the first days and weeks post-LT. It is known from a large number of other conditions that a profound Se deficit constitutes a highly relevant but addressable risk factor for poor prognosis, e.g., in relation to cardiovascular disease, cancer at different sites, infections including sepsis and the current COVID pandemic, or major trauma or other severe disease [64,65,66]. Among the underlying molecular pathways with potential relevance for poor survival under conditions of severe Se deficits are disrupting effects on the immune system [67], poor control of thyroid hormone metabolism [65] and an impaired antioxidative defense potentially causing damage and cell death from ferroptosis [22,23]. However, clinical experiences with supplemental Se are sparse, and only few trials have reported consistent and convincing results, e.g., in relation to long-term survival, avoiding endemic Se-related diseases such as Kashin–Beck and Keshan disease, endocrine Graves’ orbitopathy or improving vaccination success [66,68]. In light of the profoundly low Se status observed in the LT patients, an adjuvant Se supplementation, potentially along with other essential micronutrients, should be considered both before and after LT. From all experiences with large scale Se supplementation studies, daily dosages of around 200 µg can be considered as safe and free of unintended side-effects, whereas 800 µg per day would constitute the upper tolerable limit, obviously chosen carefully, as the first toxic symptoms were observed at higher intakes of several mg Se per day in a clinical setting [69].

Among the particular strengths of our study are the relatively high number of patients undergoing LT, and the consequent longitudinal monitoring of Se status during the intervention and the immediate time span around surgery and thereafter. Moreover, the full assessment of all three established biomarkers of Se status by validated tests provides solid information on Se status and its metabolism and dynamic alterations. Among the limitations are the nature of the study as a purely observational analysis, constricting our insights to associations only, and the lack of tissue biomarkers from biopsies, which would allow further insights into the cellular processes and intracellular Se status and hepatic Se metabolism.

## 5. Conclusions

Patients undergoing LT display considerable deficits in Se and selenoproteins before, during and after organ replacement. Biomarkers of Se status show notable correlation to liver pathology scores and prognosis, and may be monitored in order to guide adjuvant Se supplementation, which should be considered in order to improve overall health, convalescence and prognosis. However, currently, respective supplementation studies are missing, and the notion—albeit well founded on scientific grounds—lacks experimental proof from clinical trials.

## Figures and Tables

**Table 1:** Anthropometric and clinical characteristics of patients with hepatocellular carcinoma (HCC) at the time of surgery.

	Alive (*N* = 63)	Death (*N* = 16)	Total (*N* = 79)	*p* value
Sex				0.39
female	10 (15.9%)	4 (25.0%)	14 (17.7%)	
male	53 (84.1%)	12 (75.0%)	65 (82.3%)	
Age				0.79
Median (IQR)	59 (31, 70)	60 (51, 72)	59 (31, 72)	
BMI				0.88
Median (IQR)	27 (18, 39)	27.0 (22, 43)	27 (18.0, 43)	
MELD Score *				0.24
Median (IQR)	11 (6, 25)	13 (7, 32)	11 (6, 32)	
Child–Pugh Score				0.26
A	40 (63.5%)	8 (50.0%)	48 (60.8%)	
B	15 (23.8%)	7 (43.7%)	22 (27.8%)	
C	8 (12.7%)	1 (6.3%)	9 (11.4%)	
Transplantation indication				0.80
EtOH	29 (46.0%)	8 (50.0%)	37 (46.8%)	
HCV	21 (33.3%)	4 (25.0%)	25 (31.7%)	
other **	13 (20.6%)	4 (25.0%)	17 (21.5%)	
Pre-OP Neutrophiles				0.33
Median (IQR)	2.8 (1.3, 22.3)	3.3 (1.5, 16.2)	3.0 (1.3, 22.3)	
Pre-OP Lymphocytes				0.63
Median (IQR)	1.1 (0.3, 3.1)	1.1 (0.6, 2.4)	1.1 (0.3, 3.1)	
Pre-OP NLR ***				0.69
Median (IQR)	2.9 (0.5, 30.2)	3.2 (1.3, 12.4)	3.0 (0.5, 30.2)	
TSH				0.63
Median (IQR)	1.90 (0.04, 6.76)	1.47 (0.09, 10.76)	1.90 (0.04, 10.76)	
Pre-OP Se				0.10
Median (IQR)	56.6 (14.9, 99.5)	48.8 (32.7, 89.0)	55.4 (14.9, 99.5)	
Pre-OP SELENOP				0.18
Median (IQR)	2.07 (0.64, 4.66)	1.98 (1.01, 3.32)	2.06 (0.64, 4.66)	
Pre-OP GPx3				0.15
Median (IQR)	276.0 (40.7, 356.3)	258.5 (90.5, 363.3)	269.3 (40.7, 363.3)	

* MELD; Model for End-Stage Liver Disease, ** other; including HBV, NASH, cryptic, heamatochromatosis, *** NLR; neutrophiles to lymphocytes ratio.

**Table 2:** Specifics of the predictive models used.

	Model I	Model II	Model III	Model IV	Final Model
Lymphocytes			0.23		0.38
			[−0.13, 0.60]		[−0.07, 0.83]
TSH				0.40 *	0.52 *
				[0.05, 0.75]	[0.11, 0.94]
MELD Score		0.63 **			0.61 **
		[0.25, 1.02]			[0.20, 1.02]
Se	−0.62 *				−0.66 *
	[−1.11, −0.13]				[−1.22, −0.10]
AIC	158.3	154.4	163.9	160.4	146.4
Pseudo R^2^	0.07	0.10	0.01	0.05	0.23

All continuous predictors are mean-centered and scaled by 1 SD. *** *p* < 0.001; ** *p* < 0.01; * *p* < 0.05.

## Data Availability

The data presented in this study are available on request from the corresponding author. The data are not publicly available due to reasons of privacy.

## Supplementary Materials

The following are available online at https://www.mdpi.com/2072-6643/13/2/619/s1. Figure S1. Sample selection scheme and allocation to time span categories for analysis of patients undergoing liver transplantation (LT). All the analyses were conducted on the set of samples obtained after full curation of the data, yielding a set of 221 samples from 79 patients.
