# Supplementary material for: Serum Selenium Status as a Diagnostic Marker for the Prognosis of Liver Transplantation
Source: Nutrients. 2021 Feb 14;13(2):619. doi: 10.3390/nu13020619 (PMC7918136; doi:10.3390/nu13020619)
Supplement: Supplementary file 1 [file nutrients-13-00619-s001.pdf]

nutrients

MDPI

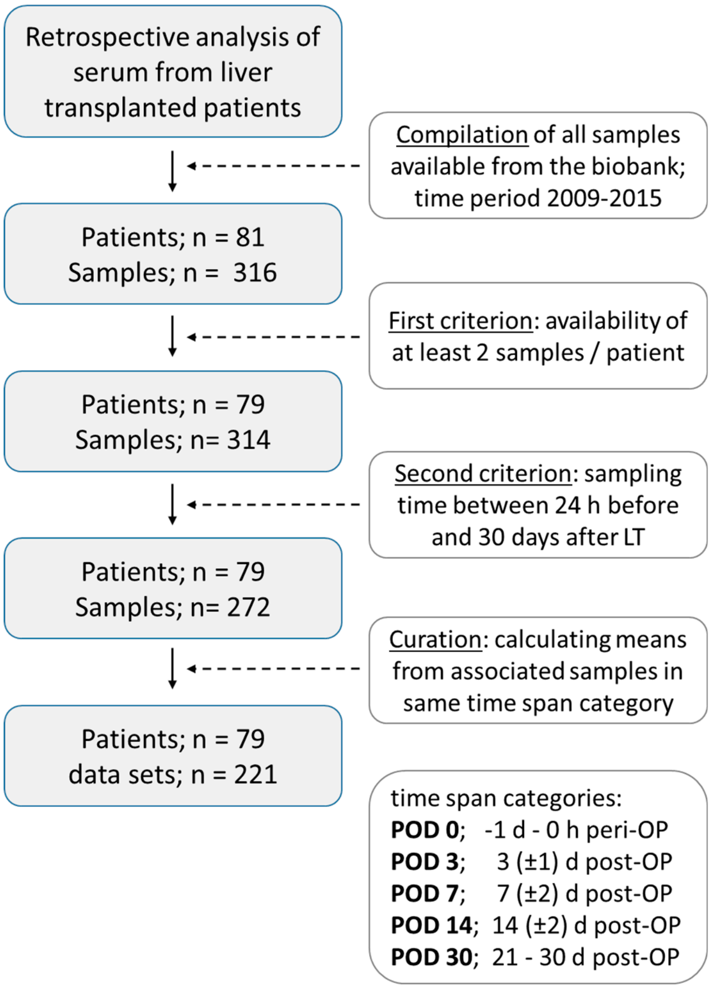

Figure S1. Sample selection scheme and allocation to time span categories for analysis of patients undergoing liver transplantation (LT). All the analyses were conducted on the set of samples obtained after full curation of the data, yielding a set of 221 samples from 79 patients.
